# Design and evaluation of a cognitive health education pilot program according to the RE-AIM framework

**DOI:** 10.1371/journal.pone.0260934

**Published:** 2021-12-03

**Authors:** Manqiong Yuan, Xiao Xiao, Yifan Wang, Yaofeng Han, Rongmu Zhang, Hanhan Fu, Ya Fang

**Affiliations:** 1 State Key Laboratory of Molecular Vaccinology and Molecular Diagnostics, Xiamen University, Xiamen, China; 2 Key Laboratory of Health Technology Assessment of Fujian Province, School of Public Health, Xiamen University, Xiamen, China; Uniwersytet Jagiellonski w Krakowie Biblioteka Jagiellonska, POLAND

## Abstract

**Objective:**

Most formats of currently used community-based health education for cognitive impairment prevention are limited to one-way communication, such as distributing leaflets, pasting posters, or holding a lecture, and they lack comprehensive evaluation. Here we aim to design, test, and evaluate a novel pilot cognitive health education program combined with psychosocial interventions (CHECPI).

**Methods:**

We designed the CHECPI program and tested it among adults aged 60 and over in an aging-friendly community in 2018. Multidimensional cognitive functions were measured by the Montreal Cognitive Assessment (MoCA) before and three months after the CHECPI program. Quantitative and qualitative analyses were performed based on the RE-AIM (Reach, Effectiveness, Adoption, Implementation, and Maintenance) framework to evaluate the program. Wilcoxon signed-rank tests were used to assess changes in multidimensional cognitive functions.

**Results:**

The CHECPI program was comprised of 12 courses and introduced 5 kinds of psychosocial interventions. Reach: 28 older adults participated in the program, of whom most were female (*n* = 22) and younger elderly with an average age of 65.32 years. Effectiveness: 19 participants finished≥6 courses as well as the follow-up survey. Although their MoCA scores did not improve significantly, they had increased their visuospatial ability significantly (with the average score increasing by 0.42). Adoption: the community officers, lecturers, and participants highly recommended the program, but they agreed that the lack of professional instructors may hinder its popularization. Implementation: the program was implemented in full accordance with the pre-program design. Maintenance: three months after the program, 17 participants had maintained at least one of the seven healthy behaviors that were introduced in the program.

**Conclusions:**

Younger female elderly were more willing to participate in the program. It enhanced participants’ visuospatial ability, but a sufficient number of professional instructors are crucial for large-scale promotion.

## Introduction

China’s population is the largest in the world and is rapidly aging. This presents China with enormous challenges. By the end of 2016, there were 231 million people aged over 60 in China, accounting for 16.7% of the total population. The Chinese elderly population is expected to exceed 300 million in 2025 and reach a peak of 487 million in 2053, when it will account for one-quarter of the global elderly population [1]. As individuals age, cognitive function gradually declines and the risk of cognitive impairment and Alzheimer’s disease (AD) increases.

Cognitive dysfunction has become one of the most urgent public health problems for aging societies. Currently, among individuals aged over 60, the prevalence of mild cognitive impairment (MCI) is 12%-18% [2] and of AD is 1.64%-7.09% [3]. Meanwhile, approximately 10%-15% of patients with MCI will progress to AD within a year [4]. Cognitive dysfunction is a chronic disease that is difficult to detect, with a low treatment rate but a high disability risk. Once it develops into AD, there is no effective treatment. Mild cognitive dysfunction is associated with many risk factors, such as unhealthy behaviors (e.g. smoking/secondhand smoking, alcohol abuse, and unhealthy diet) [5, 6], chronic diseases (e.g. obesity [7]), and psychological factors (e.g. depression [8]).

The decline of cognitive function can be slowed if the elderly are aware of cognitive impairment-related risk factors and consciously take preventive measures [9, 10]. For example, studies have suggested that physical activity, particularly aerobic exercise, and healthy behaviors such as cognitive training and good nutrition are necessary to optimize brain function and prevent cognitive decline [11, 12]. Health education, by using easily understandable language to establish a healthy mindset, is one of the most suitable ways to increase awareness of cognitive impairment prevention. Mirowsky [13] has found that the effect of health education increases with age for the elderly. Through health education, the elderly can learn exercises that enhance their sense of self-efficacy and improve their ability to control their own lives, and ultimately improve their health [14]. In the process of cognitive health education among the elderly, the key issue is to enhance their awareness of dementia prevention and establish the internal motivation to independently take preventive measures in their daily life [15].

However, the format of community-based health education for cognitive impairment prevention, in terms of programs that have actually been implemented, is currently limited to health counseling, distribution of health handbooks, poster publicity, and lectures. Briant [16] implemented a novel approach to health education through story-telling and sharing, which achieved excellent results. More new formats are urgently needed to achieve better results in health education for the elderly. There is emerging evidence for the benefits of psychosocial interventions in dementia care [17–19]. Psychosocial interventions encompass cognitive, memory, emotional and social support, which can help the target population improve outcomes such as self-confidence and social interactions.

To date, academic studies have mainly evaluated new cognitive interventions by examining their effectiveness. Although effectiveness is a crucial aspect of an intervention, its application is affected by many other factors, such as accessibility, applicability, and sustainability. There are many programs of proven efficacy that fail when implemented in real-world settings, resulting in wasted resources and unmet needs. A new health promotion or education program should be evaluated on the basis of a broad array of issues so that it can genuinely help decision-makers to make more informed judgments and effective use of scarce resources [20]. The Reach, Effectiveness, Adoption, Implementation, and Maintenance (RE-AIM) framework, proposed by Glasgow et al in 1999 [21], is a systematic evaluation method that helps us to understand a broad array of issues that an effective program must address. RE-AIM informs how to evaluate interventions that can improve the sustainable adoption and implementation of effective, generalizable, and evidence-based interventions [22]. In particular, it is useful in the evaluation of complex environment-based interventions [23, 24].

In this pilot study, we aimed to design, test, and evaluate a novel program of cognitive health education combined with psychosocial interventions (CHECPI). We designed the program by integrating content corresponding both to risk factors and to psychosocial interventions. We then tested it among older adults in an aging-friendly community in Xiamen, China. Afterward, we evaluated it based on the RE-AIM framework. The results of this study informed preliminary estimates of effectiveness and acceptability and feasibility of delivery in real-world settings.

## Materials and methods

### Formation of CHECPI

The modifiable influencing factors of cognitive decline can be summarized as comorbidities, lifestyles, and psychological factors [25, 26]. The content framework of CHECPI thus included an introduction that covered cognitive influencing factors, guidance on healthy living behaviors, and suggestions for cognitive-related disease management. The psychosocial interventions included game therapy[27, 28], art therapy [29], problem-solving therapy [30], reminiscence therapy [31], and group support therapy [32], all of which have been proven to be effective for early dementia.

### Sample

Before the CHECPI program was tested, we carried out a publicity program to recruit participants from an aging-friendly community in Xiamen, China. The recruitment notice was sent out through several channels such as a WeChat group, WeChat official account tweets, and posters at the community notice bar. People who were interested in the program participated in the publicity activities voluntarily at the community elderly activity center. The publicity activities included the introduction of the CHECPI program by posters as well as oral presentations, questionnaires, screening of cognitive function, and playing of simple cognitive games. It attracted 91 middle-aged and older adults, of whom 83 were aged over 60 years (see S1 Dataset). Since the elderly generally have relative difficulties in curriculum interactions and small class is more conducive to perform psychological interventions, we controlled the number of participants to ensure the education quality. Given that the sample sizes in cognitive intervention studies are usually less than 30 [33], we recruited CHECPI program participants on a first-come-first-served basis up to 30. Inclusion criteria were age over 60 years, ability to communicate effectively, and voluntary participation in the program. Exclusion criteria included audio-visual impairment, severe brain injury, and psychiatric disorders. In the end, a total of 28 older adults participated in the CHECPI program for at least one course, and 21 finished at least six courses (12 courses in total), of whom 19 finished the follow-up survey three months after the program (see S1 Dataset). Data of the 19 participants were used to measure the effectiveness of the program. Our study was approved by the ethical review committee of the School of Public Health, Xiamen University (SPH-XMU2015006). Informed consent was obtained from all respondents.

### Measures

Cognitive function was measured by the Montreal Cognitive Assessment (MoCA), which is widely used as a credible preliminary screening tool for mild cognitive impairment [34]. The MoCA scores ranged from 0 to 30, with a higher score indicating better cognitive performance. Six cognitive subdomains can be extracted including delayed memory (five points), visuospatial ability (four points), executive ability (four points), attention (six points), language (five points), and orientation (six points). In addition, participants’ general demographic information including age, gender, highest level of education, and marital status were also recorded. Participants’ performance in each course and the feedback on the program were recorded in a timely fashion by three regular teaching assistants, who were undergraduate students in public health and had received formal program training to guide the elderly in class discussion as well as in practicing the content of the program after class. Baseline data were extracted from the information collected in the publicity activity mentioned above, and the follow-up survey was conducted three months after the program.

### Analytical strategy

The RE-AIM framework was used to evaluate the *Reach*, *Effectiveness*, *Adoption*, *Implementation*, and *Maintenance* of this pilot program. Referring to original definitions, we interpret the *Reach* as the absolute number and basic demographic information of those who were attracted by the program publicity activity and persisted in participating in the program. The *Effectiveness* was measured by the changes in the MoCA scores of the 19 participants who attended at least six courses and finished the 3-month follow-up survey. The score changes were assessed by a Wilcoxon signed-rank test. The *Adoption* was evaluated by the opinions of relevant participants and personnel gathered by semi-structured qualitative interviews. We interpreted the *Implementation* by the degree of program completion. The *Maintenance* was measured by the retention rate of the seven kinds of healthy behaviors that had been introduced in the CHECPI courses.

## Results

### CHECPI

The program consisted of 12 courses, which were conducted once a week with application of the five types of psychosocial interventions. Following the example of the existing literature, we included living habits, comorbidity, and mental health as intervention factors. Table 1 displays the teaching content and psychosocial interventions that were performed. Each course consisted of a 45-minute lecture, followed by a 15-minute psychosocial intervention. Participants were divided into three groups according to their personal preference to comply with the rules of group support therapy. For each group, a teaching assistant was assigned to guide the participants in class discussion as well as in practicing the teaching content after class. Psychosocial interventions were combined with course content in many ways that enhanced classroom interaction and improved the enthusiasm of the participants.

**Table 1:** Curriculum of cognitive health education combined with psychosocial interventions.

Order	Lecture topic	Psychosocial Interventions applied	Specialty
1	Introduction of cognitive impairment	Group support therapy	Establishing group contacts and discuss favorite performances
2	How does the brain work?	Game therapy	Clapping hands in accordance with rhythms
3	Geriatric nutrition and cognitive related nutrient	Game therapy	Group recipe design
4	How to exercise the brain in normal times	Game therapy	Knitting Chinese knots
5	Abdominal aspiration and sleep guidance	Art therapy	Listening to music and meditate
6	The importance of quitting smoking and Limiting Alcohol	Art therapy	Creating a rap for quitting smoking and recite it
7	Identification and use of health products	Game therapy	Brain twists and words guessing
8	Physical exercise suitable for old people	Group support therapy	Learning Baduanjin exercise^a^ by group
9	How to be positive and feel happy	Problem solving therapy	True story sharing
10	Prevention of Cardiovascular and Cerebrovascular Diseases	Group support therapy	Group cardiopulmonary resuscitation and first aid training
11	Prevention of diabetes mellitus	Art therapy	Drawing for yourself
12	Recall the past, look into the future	Reminiscence therapy	Looking for memories of old Xiamen
Final Party	Knowledge Competition and Joint Performance	Combination of all	Let the elderly enjoy the stage and build self confidence

^a^Baduanjin exercise: a traditional Chinese health-promoting exercise.

### RE-AIM evaluation

#### Reach

The program publicity activity attracted 91 middle-aged and older adults, of whom 83 were aged over 60 years. Among the 83 respondents, the oldest was 77 and the mean age was 65.61. Most of them were female (*n* = 58) and married (*n* = 72). We then recruited participants on a first-come-first-served basis, resulting in a total of 28 older adults participating in at least one course (12 courses in total), with an average attendance of 8.04 courses. As shown in Table 2, there were no statistical differences in age, MoCA score, gender, marital status, or highest level of education between participants and non-participants. The average course participations for the 28 participants was 7.07 (ranged from 1 to 12). Among them, 21 finished at least half of the courses and 18 finished at least eight courses. Four older adults participated in all 12 courses, and five participated in 11 courses. Overall, most of the participants were younger elderly with an average age of 65.32 years, and most were female (*n* = 22).

**Table 2:** Basic information of the 83 older adults attracted by the program publicity activity.

Variables	Participants (*n* = 28)	Non-participants (*n* = 55)	χ^2^ /*t*^a^	*p*
Age, mean±SD	65.32±4.65	65.76±4.10	0.444	0.658
MoCA, mean±SD	24.79±2.71	22.98±5.94	-1.897	0.061
Gender, n(%)			1.517	0.218
Female	22(78.57)	36(65.45)		
Male	6(21.43)	19(34.55)		
Marital status, n(%)			0.000^b^	1.000^b^
married	24(85.71)	48(87.27)		
Others	4(14.29)	7(12.73)		
Highest level of education, n(%)^c^			4.295	0.117
<6	7(25.00)	14(25.45)		
6–9	5(17.86)	21(38.18)		
>9	16(57.14)	20(36.36)		

^a^t-test for continuous variables: age and MoCA. Chi-square test for categorical variables: gender, marital status, and highest level of education.

^b^Continuity Correction Chi-square test.

^c^ Highest level of education: years of formal education received.

#### Effectiveness

As shown in Table 3, the average MoCA score of the 19 participants was 26.05±2.46 at the initial baseline, and slightly increased in the follow-up survey after three months (26.26±2.84). Although the MoCA score did not improve significantly (*z* = 0.209, *p* = 0.431), participants did increase their visuospatial ability (*z* = -1.903, *p* = 0.045). The other five cognitive subdomains did not show statistically significant changes (*p*>0.05).

**Table 3:** Wilcoxon signed-rank test of MoCA scores between baseline and 3-month follow-up survey.

	Mean±SD		*z*	*p*
	Baseline	3-month follow-up		
MoCA total score	26.05±2.46	26.26±2.84	-0.209	0.431
delayed memory	3.58±1.39	3.84±1.26	-0.276	0.388
visuospatial abilities	3.16±0.96	3.58±0.61	-1.903	0.045
executive ability	3.42±0.84	3.58±0.77	-0.879	0.222
attention	5.84±0.38	5.74±0.45	-1.000	0.314
language	4.11±1.15	3.63±1.42	-1.130	0.136
orientation	5.95±0.23	5.90±0.46	-1.000	0.498

#### Adoption

The interviews were semi-structured with questions concerning opinions on the best part of the program, obstacles, and advice for long-term implementation of the program. 2 community officers, 5 lecturers/teaching assistants, and 25 participants were interviewed after the CHECPI program. All interviews were conducted face-to-face and lasted 10–15 minutes. At the organizational level, the community officer expressed a high willingness to adopt the program, but the fact that implementation of the program was restricted by the lack of professionals was their main concern. At the individual level, it was difficult for the lecturers to carry out the program over an extended time because of busy work schedules or traffic issues. The older adults believed that the program could improve their health, but occasionally there were time conflicts that forced them to be absent from the courses. Some specific interview contents and feedback are shown in Table 4.

**Table 4:** Responses of key informant interviews with program related personnel.

Objects	What was the best part of the program?	Obstacles to Long-term implementation of the program	What is some advice for the implementation of the program?
Community Officers *n* = 2	“Concern about cognitive health is great.” “Games and art teaching are very suitable for the elderly.”	“Such a program requires great enthusiasm of the organization staff.” “There are many elderly group activities in the community which need to be organized, so it is difficult to allocate manpower to cooperate with this project.”	“Our community covers a large area. The program objects are scattered in every corner of the community, which leads to lack of cohesion. We can teach the existing health escorts in the community and let them help other old people.”
Lecturers and teaching assistants *n* = 5	“Contact with healthy elderly people in the community helps me understand the importance of diseases prevention.” “It’s a good attempt to combine art with medicine.”	“I’m a doctor and I’m too busy at work, but I think community health education is also very important.” “This community is too far away from me. I’m afraid it’s not easy to come here regularly.” “I have to raise my grandson. So I don’t have much time for class.”	“The frequency of the course should be kept once a week, and the lecture time should be controlled within half an hour.”
			“The elderly have weakness in learning professional knowledge. The course should be more interactive with the elderly, let the elderly learn by themselves.”
Participants *n* = 25	“It’s interesting to knit Chinese knots.” “I’m in a better state of mind after class.”	“I’m in geriatric university. You should arrange your time reasonably and not conflict with my university curriculum.”	“The course content should be recorded in a book for them to review.” “Practicing singing and dancing before class rather than after class helps to improve our attention in class.”
	“Develop the habit of listening to music improve my sleep.”	“We want to see more experts come to give us lectures, but it seems difficult to invite them.”	“The course should teach more about geriatric nutrition, and chronic diseases management.”

#### Implementation

**Fig 1:**
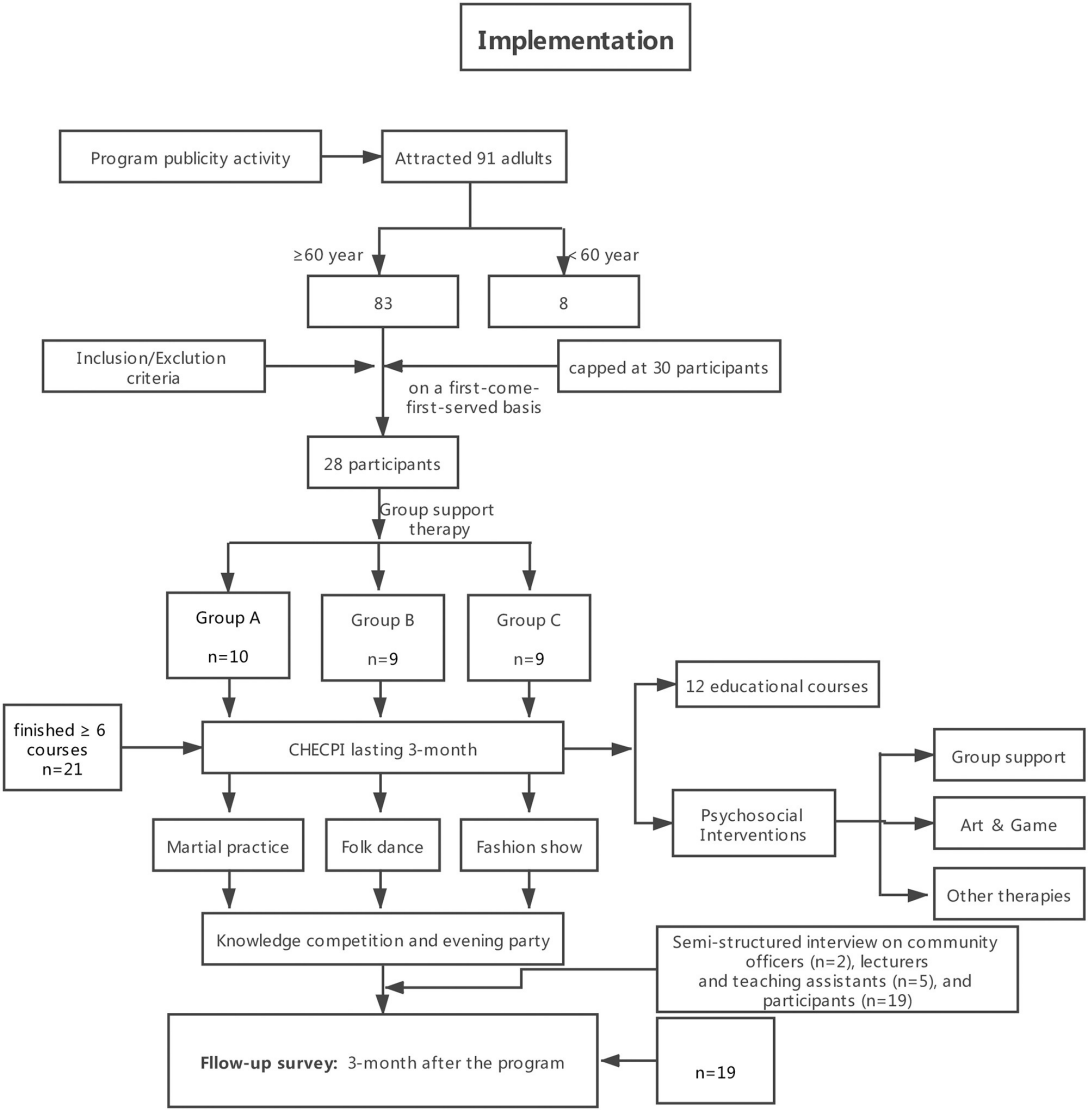
Implementation of cognitive health education combined with psychosocial interventions (CHECPI). Each course consisted of content teaching (45 mins) and different kinds of psychosocial interventions (15 mins). Martial Arts Practice, Folk Dance, and Fashion Show were the performances prepared by different groups for the final party.

The implementation process of this pilot program is shown in Fig 1. The program was carried out in full accordance with the scheduled plan. The plan was to recruit 30 participants, and 28 participants were recruited in the end. The 28 participants were classified into three groups in accordance with the practices of group support therapy. Art therapy, game therapy, and problem-solving therapy were also applied in the 12 courses. At the end of the program, we held a knowledge competition and an evening party to celebrate the completion of the project. The community provided a well-equipped classroom for implementing the program, but some of the participants had to walk a substantial distance from their homes in order to attend. This resulted in some absences during bad weather.

#### Maintenance

In the follow-up survey that was performed three months after the CHECPI program, we asked the participants about their retention of the seven kinds of healthy behaviors introduced in the CHECPI program. This was assessed by the question, “Have you developed the following behaviors according to the content of the CHECPI courses?” Three options were provided (*Yes*, *No*, and *Unknown*). The behaviors included doing finger exercises, ingesting foods that are good for the brain, practicing abdominal breathing, doing Baduanjin exercise (a traditional Chinese health-promoting exercise), communicating with others frequently, being willing to meet new friends, and participating in group activities. Among the 19 participants, 17 had maintained at least one type of the behaviors described above, and 6 had maintained more than two types. However, some of the participants reported that they had almost forgotten the teaching content and needed a review.

## Discussion

We introduced psychosocial interventions as part of the design of a novel cognitive health education program for the elderly and tested it in an aging-friendly community. The RE-AIM framework was adopted to evaluate the program both qualitatively and quantitatively. This pilot program was shown to have a positive effect on the visuospatial abilities of its participants. However, as it is a small pilot study, a larger sample size in a randomized controlled trial was needed to establish the efficacy, and the program also needs to be adapted and refined to obtain more desired effects.

The *Reach* of the program showed that younger female elderly were more willing to participate in both the program publicity activity and the courses. This has also been the case in many prior community-based cognitive intervention studies [35–37]. For example, an imagery thinking training study recruited 123 community-dwelling older participants, of whom 108 (87.80%) were women and 80 (65.04%) were younger than 70 years [35]. Even for the interventions based on chess and card intelligence games, which appear to be more popular among men, there were still more female participants than male (78 v.s. 39) and 83.8% were younger than 75 years [36]. Moreover, even in a large-scale cognitive training program that recruited 2,832 volunteer samples, 75.87% of the participants were women. Roy [37] also found women were more likely to participate in social activities than men. To improve the representativeness, more activities that men are interested in can be introduced to keep the participant population homogeneous with regard to gender in future intervention programs.

Although the average MoCA score increased in the follow-up survey, the change was not statistically significant. In retrospect, this is not surprising, and there are several possible explanations: (1) health education interventions help participants to acquire and maintain knowledge, and guide them to put this knowledge into practice. Therefore, the effects would proceed in stages: an increase in knowledge of what to do, behavioral changes, and translation of the behavioral changes into actual performance changes [38]. As a result, the participants may need more time to show any effects on cognitive function. (2) For older adults, cognitive function declines with increasing age. In this study, the average MoCA total score increased slightly, though not significantly, which may indicate beneficial effects in slowing cognitive decline. However, this effect could not be proven due to the absence of a control group. (3) Prior studies have suggested a moderate effect of intervention in cognitive impairment that requires a sample sized ≥26 subjects per group to detect an effect of intervention with 80% power at *p*<0.05 [39, 40]. However, only 19 valid participants ultimately remained and therefore the sample size in this study might not be sufficient to detect a modest change.

In recent years, there have been many programs aimed at improving cognitive function that have mainly focused on cognitive training, aerobic exercise, and health education at home and abroad. Most studies have shown that cognitive training and aerobic exercise can improve cognitive function [41, 42]. However, health education is usually used as a control intervention or as part of a joint program, and its effect is rarely evaluated. Health education encourages people to change their behaviors by disseminating health knowledge. It plays an important role in the prevention of health problems. The complex health problems of the elderly make them pay more attention to health knowledge, but existing health education for the elderly is not tailored to individual needs. It therefore lacks pertinence and does not have the desired effect. Studies showed that most of the elderly lacked knowledge of dementia, and therefore there is a need for comprehensive cognitive health education [43]. It is meaningful to adopt various formats of cognitive health education that enable the elderly to acquire dementia-related knowledge while continuously activating the brain, so as to achieve better prevention of AD.

The program was carried out strictly according to plan, but there were some emergency situations such as a sudden change of course time due to bad weather. Some participants were hard to reach by WeChat, resulting in absences from those course sessions. In the future, we can establish a group leader to make emergency notifications. Community officers gave a high appraisal of the program and were willing to extend it to other communities. However, it was recognized that the lack of professional health educators may limit the success of the program.

We designed the CHECPI program, a combination of psychosocial interventions and cognitive health education, and evaluated it based on the RE-AIM framework. Some limitations should be acknowledged. First, we conducted the pilot program in an aging-friendly community that had available support from the officers and a well-equipped classroom. Whether the program is suitable for communities with inadequate cooperation remains to be studied. Second, due to the absence of a control group, we could not evaluate the effect of the psychosocial interventions. Different psychosocial interventions might be of benefit to the elderly to different extents, which can be studied in the future for the improvement of the program. Third, due to the lack of a long-term follow-up survey, we cannot evaluate the maintenance of the CHECPI program. Fourth, the sample size was small in this pilot study, and thus the results provided an preliminary estimate of effectiveness that should be tested in a larger trial.

## Supporting information

S1 Dataset Dataset of 83 respondents and 19 valid educated participants. (XLSX)
